# Caregivers perception of the implementation of an ICU diary

**DOI:** 10.1186/2197-425X-3-S1-A653

**Published:** 2015-10-01

**Authors:** S Cabon, AS Debue, J Charpentier, N Asselie, C Brunie, N Ericher, J Garcia, L Gidel, H Lanclas, A Pileyre, JP Mira, F Pène

**Affiliations:** Hopital Cochin, Assitance Publique Hôpitaux de Paris, Paris, France; Hôpital Cochin, Paris, France

## Introduction

Prolonged stays in ICU generate anxiety for vulnerable patients and family members and are often associated with PTSD. The use of an ICU diary in which families and caregivers write about the milestones of the patient's stay in the ICU has been proposed as an aid to fill in gaps in patients' memories. Still, caregivers have not widely adopted the keeping of ICU diaries and provision of an ICU diary remains a rare event in European ICUs.

## Objectives

We conducted a survey to assess caregivers expectations or fears and identify potential barriers to the implementation of an ICU diary project in our medical ICU.

## Methods

A multidisciplinary working group has built a program to implement ICU diaries in our 24-bed MICU. Main steps involved i) consultation with ICU teams and experts in social sciences familiar with ICU diaries, ii) definition of patient diary guidelines to ensure all participants write in a similar way, iii) information sessions to explain principles and roles of an ICU diary to the entire ICU staff. Eight weeks before the implementation of ICU diaries in the unit, we designed and distributed a 22-item survey to evaluate the caregivers perception of the program and understand potential sources of concern before implementation, as well as 5 questions meant to collect opinions on practical aspects of the implementation of the project.

## Results

Out of the 104 staff members, 57 only completed the survey (RN = 25, aid-nurses = 15, MDs = 13, other = 4) among which 52 had participated to one of the 3 information meetings previously organised. Altogether, 80% of respondents identified the ICU diary as a potentially helpful tool to improve patient psychological outcomes and family satisfaction, whereas 61.4 % also identified potential benefits for caregivers. The ICU diary was perceived as a useful tool for communication with the patient and its family for respectively 66% and 49% of respondents. Thirty-three per cent of caregivers replied that filling the ICU diary should be part of routine care. Increased workload (53%) and potential breach in medical confidentiality (46%) were the obstacles most commonly perceived by the respondents. Only 32% of caregivers expressed concerns related to the implementation of the diary.

## Conclusions

Conducted in a program aimed to involve caregivers and facilitate the implementation of an ICU diary, this survey reveals a positive attitude of respondents toward ICU diaries and adequate perception of potential benefits for patients and families. The fair response rate (50%) is the main limitation of our study. Data related to the perception of caregivers 6 months after the implementation of ICU diaries in our MICU will also be presented.

## References

[1] Jones C (2012). Intensive care diaries and relatives' symptoms of posttraumatic stress disorder after critical illness. American Association of Critical-Care Nurses.

